# Draft genome sequence of *Methanocalculus natronophilus* sp. strain Z-7105^T^, an alkaliphilic, methanogenic archaeon isolated from a soda lake

**DOI:** 10.1128/mra.00350-24

**Published:** 2024-06-04

**Authors:** Sabrina M. Elkassas, Margrethe H. Serres, Derrick Richardson, Tatyana N. Zhilina, Julie A. Huber

**Affiliations:** 1 Department of Marine Chemistry and Geochemistry, Woods Hole Oceanographic Institution, Woods Hole, Massachusetts, USA; 2 Department of Earth, Atmospheric, and Planetary Sciences, Massachusetts Institute of Technology, Cambridge, Massachusetts, USA; 3 Department of Atmospheric and Ocean Sciences, University of California Los Angeles, Los Angeles, California, USA; 4 Winogradsky Institute of Microbiology, Federal Research Center of Biotechnology of the Russian Academy of Sciences, Moscow, Russia; DOE Joint Genome Institute, Berkeley, California, USA

**Keywords:** alkaliphile, methanogen, hydrogenotrophic, soda lake

## Abstract

A methanogenic archaeon was isolated from bottom sediments in the vicinity of Lake Tanatar II (Altai, Russia), an alkaline soda lake. Here we present the draft genome sequence of *Methanocalculus natronophilus* sp. strain Z-7105^T^.

## ANNOUNCEMENT

*Methanocalculus natronophilus* sp. strain Z-7105^T^ is an alkaliphilic, hydrogenotrophic, and halotolerant methanogenic archaeon isolated from bottom sediments in the vicinity of soda lake Tanatar II (51.65189 N,79.829406 E, Kulunda Steppe region, Altai, Russia) (1, 2). It was originally isolated at 35°C in *Natroniella* medium (DSMZ medium 784) with a headspace of pure H_2_ (DSMZ medium 784-a). The isolate was acquired from the DSMZ-German Collection of Microorganisms and Cell Cultures GmbH with isolate identifier DSM 25006. Within the methanogens, little is known about genomic or physiological adaptations to high pH; thus, the genome of *Methanocalculus natronophilus* sp. strain Z-7105^T^ was sequenced to understand how these ancient autotrophs are adapted to this pH extreme.

For DNA extraction, cells were grown anaerobically in 115-mL serum vials with 30 mL of medium. Prior to inoculation, the medium was reduced with 2.1 mL 2.5% Na_2_S × 9H_2_O and amended with 2.8 mL of Wolin’s vitamin solution (3). Vials were topped off with a headspace of 80% H_2_/20% CO_2_ after inoculation. Cultures were incubated at 35°C for 12 days.

Genomic DNA was extracted from a single culture using the Qiagen DNeasy Ultraclean Microbial Kit, following the manufacturer’s instructions. The Illumina library was prepared at the sequencing center using the Accel-NGS 2S Plus DNA Library Preparation Kit/MiSeq nanokit v2 following the manufacturer’s instructions. DNA was sequenced using short-read (Illumina MiSeq, PE 250 bp) sequencing technologies with a sequencing depth of 75×, resulting in 2,409,898 reads. Post-processing bioinformatic analyses were conducted on the Department of Energy Systems Biology Knowledgebase (KBase) (4). Default parameters were used for all analysis software. Reads were trimmed using Trimmomatic v0.36 (5), resulting in 97.7% of paired reads surviving. Reads were quality checked using FastQC v0.12.1 (6), with 0 sequences flagged as poor quality. The trimmed reads were assembled using SPAdes v3.15.3 (7), and the assembly was quality checked using Quast v4.4 (8), resulting in 179 total contigs, an N_50_ value of 273,644 bp, a maximum contig length of 403,126 bp, a total length of 2,028,189 bp, and G + C content of 51.25%. CheckM v1.0.18 (9) was used to determine a genome completion of 100% and contamination of 3.65%. Sample metadata can be found in Table 1.

**TABLE 1:** Metadata for *Methanocalculus natronophilus* sp. strain Z-7105^T^

Parameter	Data
Environmental data	
Geographic location	
Region	Russian Federation Altai, Kulunda Steppe region, Tanatar soda lake II
Geographic coordinates	51.65189 N,79.829406 E
Collection date	06/2008
Biome	Alkaline soda Lake
Lake name	Tanatar II
Sample type	Bottom sediments
Sampling method	Water collector
pH	10.4
Total mineralization	60 g/L
Growth conditions	DSMZ 784 a
Sequencing	
RNA/DNA quantification instrument	Nanodrop UV/visible spectrophotometer (Thermo Fisher Scientific)
Illumina library	Accel-NGS 2S Plus DNA library preparation kit/MiSeq nanokit v2
Sequencing technologies	Illumina MiSeq
No. of Illumina reads	2,409,898
Sequencing center	Microbial Genome Sequencing Center, University of Pittsburgh, Pittsburgh, PA, USA
Sequencing depth	75 x
Assembler	SPAdes v3.15.3
No. of contigs	179
Largest contig (bp)	403,126
Contig N50 (bp)	273,644
ORF caller	Prodigal v2.6.3
Genomic features	
Genome size (bp)	2,028,189
G + C content (mol%)	0.5125
No. of protein-coding genes	1,968
No. of RNA genes	47
No. of rRNA genes	2
No. of 5S rRNA genes	1
No. of 16S rRNA genes	0
No. of 23S rRNA genes	1
No. of tRNA genes	45
No. of other RNA genes	2
No. of CRISPR loci	2

**Fig 1:**
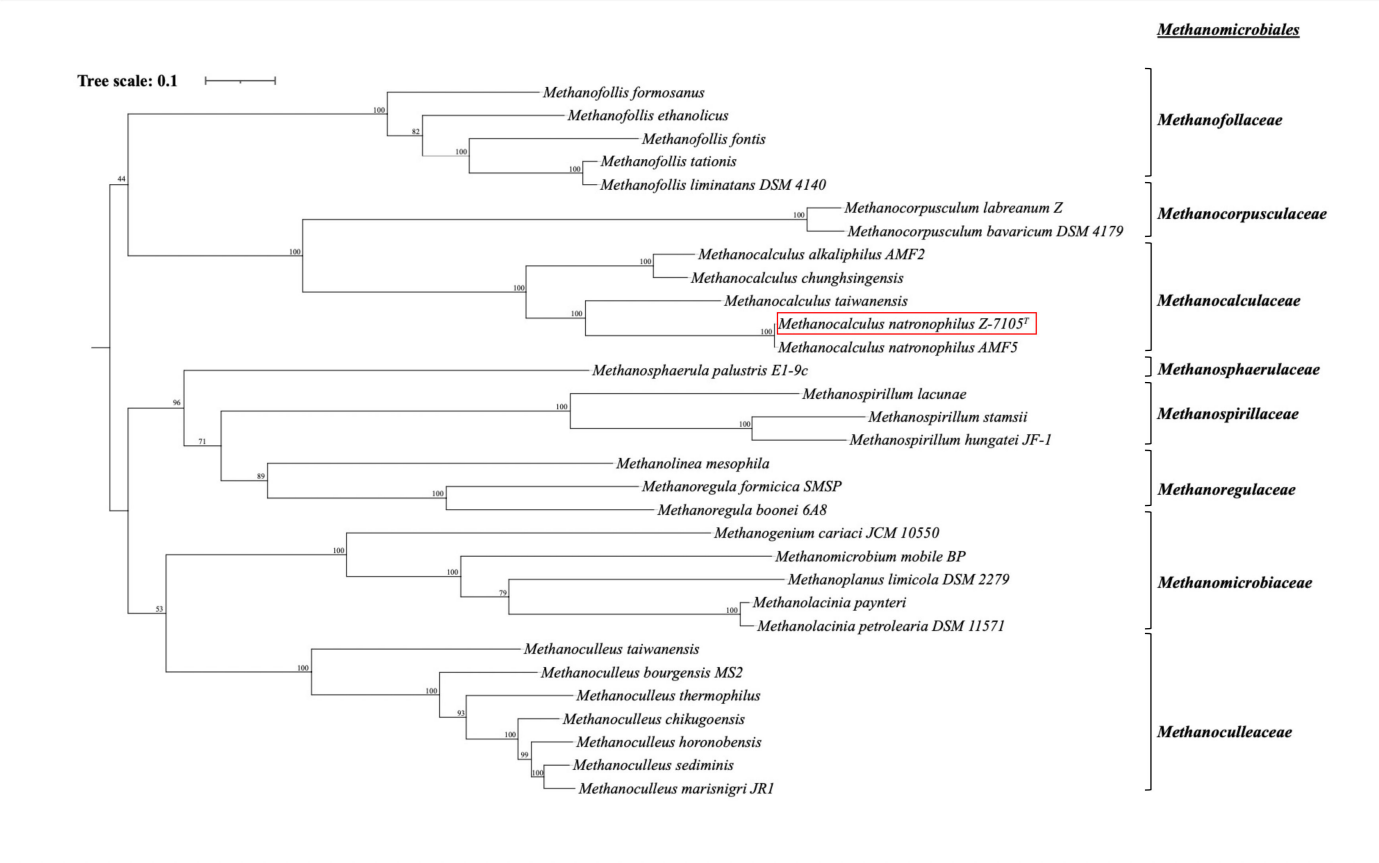
Phylogenomic tree of all RefSeq genomes of cultivated representatives of the order *Methanomicrobiales* and the position of *Methanocalculus natronophilus* sp. Strain Z-7105^T^ (in red box) within that order. The tree was generated using maximum-likelihood estimates based on alignments of nucleotide sequences within GToTree (10). Bootstrap values above 40 are shown.

Assembled contigs were annotated using Prodigal v2.6.3 (10) within DRAM v0.1.2 (11), resulting in 1,968 protein-coding genes predicted, 2 complete rRNA genes, 45 tRNA genes, and 2 CRISPR loci. *Methanocalculus natronophilus* sp. strain Z-7105^T^ has genes for the complete reductive acetyl-CoA (Wood-Ljungdahl) pathway for carbon fixation, methanogenesis via hydrogen oxidation, and formate utilization and multiple genes for proton transport, including the multicomponent Na+/H+ antiporter and V/A-type H+-transporting ATPase, presumably to maintain cell homeostasis in its alkaline environment. A phylogenomic tree constructed with GToTree v1.4.5 (12) with all RefSeq genomes from members of the order *Methanomicrobiales* is shown in Fig. 1. *Methanocalculus natronophilus* sp. Z-7105^T^ is most closely related to another representative of *M. natronophilus*, strain AMF5.

## Data Availability

Raw sequence reads for the genome of *Methanocalculus natronophilus* sp. strain Z-7105^T^ are available at NCBI under BioProject accession number PRJNA1089169, BioSample accession number SAMN40528436, and SRA accession number SRR28374704. Assembled contigs are available under accession number JBCEXH010000000.

## References

[1] Zhilina TN, Zavarzina DG, Kevbrin VV, Kolganov TV. 2013. Methanocalculus natronophilus sp. nov., a new alkaliphilic hydrogenotrophic methanogenic archaeon from a soda lake, and proposal of the new family Methanocalculaceae. Mikrobiologiia 82:681–690. doi:10.1134/S0026261713060131 25509406

[2] Samylina OS, Kosyakova AI, Krylov AA, Sorokin DY, Pimenov NV. 2024. Salinity-induced succession of phototrophic communities in a Southwestern Siberian soda Lake during the solar activity cycle. Heliyon 10:e26120. doi:10.1016/j.heliyon.2024.e26120 38404883 PMC10884861

[3] Wolin EA, Wolin MJ, Wolfe RS. 1963. Formation of methane by bacterial extracts. J Biol Chem 238:2882–2886. doi:10.1016/S0021-9258(18)67912-8 14063318

[4] Arkin AP, Cottingham RW, Henry CS, Harris NL, Stevens RL, Maslov S, Dehal P, Ware D, Perez F, Canon S, et al.. 2018. KBase: the United States department of energy systems biology knowledgebase. Nat Biotechnol 36:566–569. doi:10.1038/nbt.4163 29979655 PMC6870991

[5] Bolger AM, Lohse M, Usadel B. 2014. Trimmomatic: a flexible trimmer for illumina sequence data. Bioinformatics 30:2114–2120. doi:10.1093/bioinformatics/btu170 24695404 PMC4103590

[6] Lo C-C, Chain PSG. 2014. Rapid evaluation and quality control of next generation sequencing data with FaQCs. BMC Bioinformatics 15:366. doi:10.1186/s12859-014-0366-2 25408143 PMC4246454

[7] Bankevich A, Nurk S, Antipov D, Gurevich AA, Dvorkin M, Kulikov AS, Lesin VM, Nikolenko SI, Pham S, Prjibelski AD, Pyshkin AV, Sirotkin AV, Vyahhi N, Tesler G, Alekseyev MA, Pevzner PA. 2012. SPAdes: a new genome assembly algorithm and its applications to single-cell sequencing. J Comput Biol 19:455–477. doi:10.1089/cmb.2012.0021 22506599 PMC3342519

[8] Gurevich A, Saveliev V, Vyahhi N, Tesler G. 2013. QUAST: quality assessment tool for genome assemblies. Bioinformatics 29:1072–1075. doi:10.1093/bioinformatics/btt086 23422339 PMC3624806

[9] Parks DH, Imelfort M, Skennerton CT, Hugenholtz P, Tyson GW. 2015. CheckM: assessing the quality of microbial genomes recovered from isolates, single cells, and metagenomes. Genome Res 25:1043–1055. doi:10.1101/gr.186072.114 25977477 PMC4484387

[10] Hyatt D, Chen G-L, Locascio PF, Land ML, Larimer FW, Hauser LJ. 2010. Prodigal: prokaryotic gene recognition and translation initiation site identification. BMC Bioinformatics 11:1–1. doi:10.1186/1471-2105-11-119 20211023 PMC2848648

[11] Shaffer M, Borton MA, McGivern BB, Zayed AA, La Rosa SL, Solden LM, Liu P, Narrowe AB, Rodríguez-Ramos J, Bolduc B, Gazitúa MC, Daly RA, Smith GJ, Vik DR, Pope PB, Sullivan MB, Roux S, Wrighton KC. 2020. DRAM for distilling microbial metabolism to automate the curation of microbiome function. Nucleic Acids Res 48:8883–8900. doi:10.1093/nar/gkaa621 32766782 PMC7498326

[12] Lee MD. 2019. GToTree: a user-friendly workflow for phylogenomics. Bioinformatics 35:4162–4164. doi:10.1093/bioinformatics/btz188 30865266 PMC6792077

